# Novel Developments to Enable Treatment of CNS Diseases with Targeted Drug Delivery

**DOI:** 10.3390/pharmaceutics15041100

**Published:** 2023-03-29

**Authors:** Axel H. Meyer, Thomas M. Feldsien, Mario Mezler, Christopher Untucht, Ramakrishna Venugopalan, Didier R. Lefebvre

**Affiliations:** 1 Quantitative, Translational & ADME Sciences, AbbVie Deutschland GmbH & Co. KG, Knollstraße, 67061 Ludwigshafen, Germany; 2 Drug Delivery and Combination Products, Development Sciences, AbbVie Inc., 1 N Waukegan Road, North Chicago, IL 60064, USA; 3 Neuroscience Discovery, AbbVie Deutschland GmbH & Co. KG, Knollstraße, 67061 Ludwigshafen, Germany

**Keywords:** central nervous system, blood brain barrier, drug delivery, degradomer, nanoparticles, exosomes, focused ultrasound, convection enhanced delivery, intracerebroventricular delivery

## Abstract

The blood-brain barrier (BBB) is a major hurdle for the development of systemically delivered drugs against diseases of the central nervous system (CNS). Because of this barrier there is still a huge unmet need for the treatment of these diseases, despite years of research efforts across the pharmaceutical industry. Novel therapeutic entities, such as gene therapy and degradomers, have become increasingly popular in recent years, but have not been the focus for CNS indications so far. To unfold their full potential for the treatment of CNS diseases, these therapeutic entities will most likely have to rely on innovative delivery technologies. Here we will describe and assess approaches, both invasive and non-invasive, that can enable, or at least increase, the probability of a successful drug development of such novel therapeutics for CNS indications.

## 1. Introduction

A major challenge in the development of treatments against central nervous system (CNS) diseases is ensuring sufficient exposure to target brain tissues to achieve the desired therapeutic effects. A disappointing 98% of small molecule drugs never reach the brain at therapeutic concentrations [1]. The situation is even worse for large-molecule drugs such as antibodies or nucleic-acid based therapeutics. For example, only 0.01–0.1% of a systemically administered dose of antibody will reach the brain, which is often insufficient to elicit a therapeutic effect [2,3]. In the case of naked nucleic acid-based therapeutics such as oligonucleotides, achieving efficient brain tissue exposure would not even be possible without the use of a carrier system [4].

The root cause is the blood-brain barrier (BBB). This complex, multi-cellular and dynamic interface between the blood circulation and the brain tightly preserves brain homeostasis. It enables passage of specific nutrients such as glucose, fatty acids, and amino acids, while at the same time blocking the passage of harmful substances [5]. In doing do, it also acts as a barrier to large therapeutic molecules.

To overcome the BBB, a wide range of delivery technologies, both invasive and non-invasive, have been developed. Receptor-mediated transcytosis (RMT), one of the most advanced, has reached the clinic and holds promise for the delivery of biotherapeutics such as antibodies or enzyme replacement therapies. Since non-invasive approaches to cross the BBB are well covered elsewhere [6], they are not included in this review.

Therapeutic modalities have become more diverse with the advent of gene therapies and degradomers, both of which may require optimized brain delivery systems tailored to their specific needs. The increasing number of gene therapies, both in development and on the market, as well as the ongoing development of degradomers make the challenge of developing efficient delivery technologies for such therapeutic modalities even more pressing.

This realization led us to look at the status of gene therapy and degradomer approaches for CNS indications and to review several delivery technologies for their potential to enable a successful CNS drug development for these types of molecules. The non-invasive approaches include nanoparticles and exosomes. We cover recent medical technology innovations for gene therapy applications and degradomers.

In the final sections, we include a brief survey of medical devices enabling drug delivery, either to the whole brain, or to more localized regions of the brain or the spine: intrathecal (IT), intra-cerebroventricular (ICV), convection-enhanced delivery (CED) and focused ultrasound (FUS).

The central theme that emerges from our review is that delivering a wide range of therapeutics to the brain requires tailored technical solutions, each optimizing the pharmacokinetic profile in brain tissues to achieve therapeutic levels. We show that a convergence between medical technology (MedTech) and pharmacology is increasingly needed to achieve efficacy while reducing side effects associated with off-target exposure.

### 1.1. Use of Gene Therapy for the Treatment of CNS Indications

Gene therapy aims at modifying a patient’s genes for disease treatment by either gene replacement, gene inactivation or by gene introduction. Such approaches can be divided into two categories, viral and non-viral approaches. Historically, approval for gene therapy-based drug products has not been a focus for central nervous system indications. Gene therapy trials have covered a wide range of indications including cancer, monogenic diseases, infectious diseases, and cardiovascular diseases. In 2017 Ginn et al. listed 2597 gene therapy clinical trials for all these indications. Only 47 covered neurological diseases comprising less than 2% of gene therapy trials [7]. This is reflected in the gene therapy products that have reached the market. Even though the first gene therapy product, Gendicíne, was approved in 2003, it took until 2016 for the first gene therapy-based CNS drug, Spinraza, to reach the market. Interestingly, the situation is starting to change. Three more gene therapies for CNS indications have reached the market since 2016, as illustrated in Table 1. Overall, four out of 16 marketed gene or cell therapy products are directed against CNS diseases.

It is worth mention mentioning that these treatments do not rely on systemic application of the gene therapy-based drugs. The commercial approval in 2016 of Spinraza^®^, an antisense oligonucleotide for the treatment of spinal muscular atrophy (SMA), and subsequent commercial approval in 2017 of Brineura^®^, a hydrolytic lysosomal N-terminal tripeptidyl peptidase for the treatment of Batten disease, indicates that pharma organizations view the benefits of intrathecal (IT) and intracerebroventricular (ICV) routes of delivery as outweighing limitations (e.g., invasiveness) in the case of niche markets for orphan drugs in the neuroscience space. The commercial approval in 2019 of Zolgensma^®^, also for the treatment of SMA, represents the first adeno-associated virus (AAV) based drug for a CNS indication [8]. This was followed in 2021 by Delytact which received conditional and time-limited marketing approval for malignant glioma in Japan. To gain more insight into what direction the field is taking we performed a survey in clinicaltrails.gov to identify gene therapy clinical trials specifically for the following neuroscience indications: Alzheimer’s Disease (AD), Parkinson’s Disease (PD), Huntington’s Disease (HD), Gaucher Disease, Lou Gehrig’s Disease (ALS), and Frontotemporal Dementia (FTD). Approximately 3750 results were identified in the search query, and from the query, 16 on-going benchmark trials. The search algorithm at clinicaltrials.gov also searches for synonyms and related terms. The executed search query included the terms ‘gene therapy’, ‘viral vector’, ‘AAV’, ‘lentivirus’, ‘antisense’, and ‘oligonucleotide’. The query identified approximately 3750 records which included related terms such as ‘gene’, ‘gene transfer’, ‘DNA therapy’, ‘therapy’, ‘treatment’, ‘therapeutic’, ‘viral vector’, ‘viral’, ‘virus’ and ‘vector’. The search results then had to be narrowed down using data filtering to identify representative gene therapy studies in the target indications.

A summary of the on-going trials with a clinical phase, associated routes of administration and study duration is given in Table 2. Interestingly, none of the clinical trials utilize systemic delivery or intra-cerebroventricular delivery as a route of administration for gene therapy assets in these indications. Given that viral vectors such as AAV stimulate the immune system after systemic application, a targeted application is a feasible way to reduce/avoid activation of the immune system. Furthermore, only AAV serotypes 8 and 9 have been reported to cross the BBB and enter the brain. Nonetheless, the example of Zolgensma shows that systemic application is possible for a CNS disease.

### 1.2. Use of Degradomers for the Treatment of Neurodegenerative Diseases

While gene therapy treatments have made it to the clinics and to the market, even for the treatment of CNS diseases, degradomers are at an earlier stage. Degradomers utilize the intrinsic protein degradation machinery of the cell to remove unwanted, overexpressed, dysfunctional, or dysregulated protein targets [13]. They exist in different forms, which include PROTACs (PROteolysis TArgeting Chimeras), employing the cellular “quality control” machinery to eliminate unwanted proteins [14], AUTACs (AUtophagy-TArgeting Chimera) [15], leading the target into the autophagy mechanism, lysosome targeting chimeras (LYTACs) [16], molecules activating the endoplasmic reticulum-associated degradation (ERAD) pathway, and molecular glues, which transform the target to be structurally modified or tagged as unwanted, and to be destroyed. The degradomers can be differentiated into hetero-bifunctional molecules, including PROTACs, AUTACs, LYTACs and ERAD-pathway targeted molecules, and monofunctional compounds, which are the molecular glues. As the published dataset on PROTACs is currently the largest, we focus on this modality for the hetero-bifunctional molecules. It is expected that the relevance of the AUTACs, LYTACs and ERADs will expand in the future.

Molecular glues mediate their efficacy through proximity-induced degradation [17]. The molecules represent proximity-inducing monofunctional agents, which can have multiple biological effects in the cell. For example, by bringing functional proteins into proximity, or changing the structure of a single protein, they can influence cell signaling, interact with the immune system, remodel chromatin, and also lead to an interaction of the protein of interest (POI) with the ubiquitination system of the cell, which leads to its degradation. For example, Thalidomide and lenalidomide mediate this effect [18], which is described in more detail below in the context of the PROTACs.

As described above, while molecular glues are monofunctional with a relatively low molecular weight, chemical PROTACs are hetero-bifunctional molecules and consist of one binding site for the protein of interest (POI), a linker, and a binding site to the functional cellular protein, which leads the POI towards degradation. Thus, they are generally relatively large, with a molecular weight ranging from 500 to 1000 Da. While they are still comparatively small compared to monoclonal antibodies or peptide or nucleotide drugs, for example, their size causes physicochemical problems, which often leads to low solubility and low permeability across biological membranes. This potentially limits utility for the treatment of CNS diseases, particularly because of the resulting low penetration across the BBB.

Structurally, PROTACs contain a binding site for a specific intracellular ubiquitin ligase or a key autophagy functional protein (LC3), a linker of different lengths and constituents, and a binding site for the POI. PROTACs and AUTACs are, therefore, hetero-bifunctional molecules that bind to the POI and link it to an E3 ubiquitin ligase (PROTAC) through reversible interaction with binding sites on either molecule. By interacting with the E3 ligase the POI is (poly)-ubiquitinated and therefore labeled to undergo intracellular protein degradation in the ubiquitin-proteasome system (UPS; PROTAC).

Current PROTACs bind primarily to Cereblon (CRBN) and von Hippel-Lindau Ligands (VHL) E3 ligases. Current efforts include identifying additional potential ligases, as the CRBL and VHL ligases are expressed ubiquitously, and thus might lead to non-specific and broad-based protein degradation in all tissues. Therefore, more selectively expressed, and more specific ligase binders might lead to a more selective degradation of the POI only in the target tissues [19].

Degradomers have a number of promising advantages over conventional small chemical entities, which include the potential for an effective knock-down of the POI, similar to gene therapy or ribonucleic acid interference (RNAi) approaches, the potential for a catalytical activity, which could alleviate the issue associated with the need to achieve continuously high concentrations of the molecule within the target tissue and cell, and the potential of a selective suppression of certain mutated POIs, which might make it possible for the functional protein allele to take over the activity.

Despite the range of targets and disease states investigated preclinically, the clinical success of hetero-bifunctional degraders is so far limited, and currently described by PROTAC examples only. Nine clinical studies sponsored by Arvinas (NCT03888612, NCT04072952), Celgene (NCT04428788), Kymera (NCT04772885), Nurix (NCT04830137), Haisco Pharmaceutical (NCT04861779), Dialectic Therapeutics (NCT04886622), EnhancedBIO (NCT04669587) and BeiGene (NCT05006716) are currently ongoing. All are in Ph. 1 or Ph. 1/2, mostly for the treatment of cancer and dermatological applications. To date the only clinically relevant study demonstrating efficacy of PROTACs in humans was published in 2020 for the treatment of cancer [20]. The number of PROTAC compounds entering Ph1 clinical trials is rapidly increasing. There are currently over 18 such molecules that are either in dosing or approved for clinical trials, with several more anticipated in 2023. In addition, there have been data releases at conferences and company updates showing data for at least three ongoing programs that have achieved proof of target degradation in humans. Therefore, the degradomer field, spearheaded by PROTACs, is rapidly evolving and sustained publication of clinical efficacy studies is expected. The theoretical potential of degraders for CNS diseases is being investigated in numerous labs. In this context PROTACs have been developed for a range of CNS diseases. Preclinically, they have been described to successfully target tau, α-syn, mutant huntingtin (mHtt), and superoxide dismutase 1 (SOD1) in cellular and animal models. Diseases which are studied in preclinical models include Alzheimer’s disease (AD), Parkinson’s disease (PD), Huntington’s disease (HD), amyotrophic lateral sclerosis (ALS), as well as glioblastoma multiforme (GBM). The advantages of hetero-bifunctional degraders described above also come with challenges for treating CNS diseases. They include the limited penetration across the BBB due to the large size and bulky structure, the non-specific degradation of the POI across the whole CNS, instead of targeted degradation in the affected brain region, the concomitant reduction of proteolytic activity in different neurodegenerative disease states [21], and the transient nature of activity, which requires continuous or multiple treatments throughout the chronic phase of the neurodegenerative disease [22].

The reports describing efficacy of hetero-bifunctional degraders in preclinical in vivo models for CNS diseases are therefore limited. The first published study evaluated peptides with hetero-bifunctional PROTAC activity in in vitro and in vivo models of AD [23]. Compound TH006, a peptide consisting of 32 amino acids, was dosed at 15 mg/kg/day for 10 days in a combination of intranasal and intravenous route of application. The PROTAC reduced cortical Tau levels, as well as Tau in the hippocampus to a lesser extent, in 3xTg-AD mice (B6; 129-Psen1tm1Mpm Tg [APPSwe, Tau P301L] 1Lfa/Mmjax). While the exposure of the compound in the brain was not directly demonstrated in the study, apparently enough TH006 reached the relevant brain structures to modulate Tau levels in the brain of the animals. Arvinas demonstrated the effective penetration of selected PROTAC^®^ degraders through the BBB. As described in a press release, their reference compounds reached brain-to-plasma ratios greater than one. As a result of brain exposure lasting longer than systemic circulation, this ratio rose to 8.9 four hours after dosing. The patent describes efficacy studies with several examples of compounds. Example four reduced total Tau after intra-hippocampal injection in a time-dependent manner in Bl6 mice. Exemplary compounds 82 and 382 demonstrated a 95% reduction of pathological Tau in TG2508 mice after 24 h at doses of 15 and 30 mg/kg, respectively, via parenteral dosing.

While the published results indicate the relevance of hetero-bifunctional degraders for the treatment of CNS disorders, the number of in vivo studies is still limited. There is also not yet a clear understanding of the potential influence of formulations for the penetration of the molecules into the brain. Further studies are required to translate the in vitro results in different cellular models into preclinical in vivo models of neurodegenerative diseases. Translation from rodents to humans is yet another critical step.

Due to their large size, their low permeability across the BBB and complex mode of action, it is not yet fully clear which systemic concentrations are needed to mediate an effect on brain targets. A clear strategy for optimization of the brain availability by rational drug design is not yet available. Therefore, besides serendipitous optimization of the compound structure, the next steps towards functional brain degradomers could be to evaluate methods to increase the systemic exposure of the compound, for example by increasing the circulating drug concentration, or to enhance the systemic half-life through specific slow-release systemic formulations, or to open or circumvent the BBB. Methods for the latter two aspects are exemplified in the present paper.

## 2. Systemic Delivery

### 2.1. Nanoparticles

Nanoparticles, such as liposomes, polymeric nanoparticles, and lipid nanoparticles (LNPs), typically have a diameter between 10 and 300 nm. They differ in size, charge, chemical composition and surface properties [24,25,26]. The first nanoformulations received approval for cancer therapy more than 20 years ago following the discovery of nanoparticles having the potential for increased accumulation in tumor tissue by extravasation through fenestrated blood vessels, a phenomenon termed enhanced permeability and retention (EPR). Several nanoparticle formulations were approved for chemotherapy, for the treatment of fungal infections, hepatitis A and end-stage renal diseases. However, so far only one formulation, Copaxone, has been approved for multiple sclerosis as early as 1996 in the United States and 2001 in the European Union (EU), and to our knowledge is one of the only approved nanoformulation for a CNS disease. Another example is Invega, an intramuscular depot approved by the EU in 2011. However, Invega does not enter the brain directly but increases residence time of the active compound in the blood. Recently, efforts have centered around LNPs, which have found use in COVID vaccinations such as Comirnaty and have proven the therapeutic applicability of these formulations for nucleic acid delivery. To this end Pfizer has entered a four-year research collaboration with Beam Therapeutics utilizing Beam’s LNP-based in vivo delivery technology to deliver messenger RNA (mRNA) for gene editing programs for rare diseases in different organs, one being the CNS.

The fact that nanoparticles have only found limited therapeutic uses, especially for the CNS [27], can be attributed to several critical factors such as physicochemical properties, biological interactions with host biofluids and overcoming physiological barriers. Especially the physicochemical properties can give rise to nanoparticle toxicity [28].

Nanoparticles for therapeutic purposes usually contain reagents generally recognized as safe (GRAS). Nevertheless, they can be immunotoxic, eliciting a variety of adverse effects including immunosuppression or immunoenhancement, which in the first case can lead to infections and tumor formation, and in the second case to autoimmunity and hypersensitivity. These effects can be elicited either by the particle itself or by the active pharmaceutical ingredient (API) encapsulated in the particle. Recognition of nanoparticles by the immune system is dependent on their physicochemical properties, their cargo as well as their external milieu such as their protein corona, whose constituents in turn are determined by the nanoparticle’s physicochemical properties. In fact, it has been reported that nonviral vectors including lipoplexes have the capability to induce similar or stronger immune reactions than viral vectors [29].

Size determines the ease with which nanoparticles enter their target organ. In general, the smaller they are, the more easily they enter their target organs. However, if nanoparticles are too small (<20 nm) they are cleared from blood via the reticuloendothelial system (RES) and the mononuclear phagocyte system (MPS) [30,31] as well as by filtration via the renal glomeruli, which both lead to a significant reduction in half-life [32,33]. One common strategy to improve half-life apart from size is coating the nanoparticle with polyethylene glycol (PEG) (stealth nanoparticles) that helps avoid recognition by the RES. The upper size limit is mostly defined by the width of the interstitial space of the target organ. If the nanoparticles get too large, diffusion through the interstitial space will be hindered and uptake into target cells inhibited.

A positive charge facilitates interaction with negatively charged cell membranes [34,35] but may trigger toxicity due to easier uptake into cells. Furthermore, positively charged particles are prone to opsonization in body fluids, leading to a corona formation, a coating of the nanoparticle surface by serum proteins, which in turn results in sequestration by the MPS. A promising approach to address the problem of corona formation by coating solid-lipidic-nanoparticles (SLNPs) with a preformulated protein corona was developed within the IMI COMPACT consortium [36,37]. In this approach SLNPs were coated with human serum albumin to which transferrin was conjugated as a targeting ligand for the BBB. This coating is meant to have two effects: First, it should provide protection from binding of serum proteins to the nanoparticles which causes random corona formation. Second, it should keep targeting to the BBB effective. If a targeting ligand is coupled directly to the surface of the nanoparticle, corona formation will mask the ligands, which will block BBB targeting. Since here the targeting ligand is coupled to the corona-mimicking serum albumin, masking of the targeting ligand by the protein corona should be avoided. In vitro studies have demonstrated that this formulation still possessed a strong ability to be taken up by microvascular endothelial cells in the presence of serum in the medium, whereas uptake of nanoparticles without this modified surface were not. In addition, the first in vivo imaging experiments have shown strong signals and long residence time in the brain, indicating an effective targeting of the BBB. Whether this signal is found in the brain or in the vasculature remains to be determined.

Special care must be taken for the release kinetics of the nanoformulation. Many nanoparticulate systems have the problem of a premature burst release of the encapsulated drug. Avoiding this is important for a successful nanoparticle for two reasons [38]. First, encapsulation of a drug can help avoid peripheral side effects. This has been demonstrated, for example with Doxycycline, whose cardiotoxic side effects have been avoided by encapsulation. Second, premature release of the drug can prevent a therapeutic effect by lowering target tissue exposure below the therapeutic threshold. In this regard, it must be kept in mind that nanoparticulate systems will alter the pharmacokinetic (PK) profile of their cargo. While encapsulation of a drug in a nanoparticle may be helpful to avoid systemic side effects elicited by the drug, the pharmacokinetics of the encapsulated drug are driven by the nanoparticle, which may be less favorable than the PK of the original drug.

In addition, nanoparticles need to cross the blood-brain barrier to reach brain tissues. This may require attachment of a targeting moiety to the surface of the particle to promote interaction with the BBB and increase transport into the brain. To this end, several studies in preclinical models of neurodegenerative diseases have shown efficacy of different cargos after packaging into brain-targeted nanoparticles. [39,40,41] Still, most nanoformulations show a biodistribution profile where most of an applied dose usually ends up in the liver. Attachment of a surface targeting moiety, in combination with corona mimicry, will increase uptake into the brain to a certain extent but will not prevent most injected nanoparticles from accumulating in the liver.

### 2.2. Nanoparticles as Potential Brain Bioavailability Enhancers for Payload Carrying Nanoparticles

Recent literature suggests that the biodistribution of nanoformulations can be altered significantly and their clearance by the liver strongly inhibited. An emerging PK-enhancing adjuvant therapy uses payload-free nanoparticles referred to as nanoprimers [42] that have the function of increasing the bioavailability of other nanoparticles, whose function is to carry a therapeutic payload (therapeutic nanoparticles). The PK boosting effect is achieved by administering the nanoprimer prior to the therapeutic nanoparticles, thereby causing a delay in the hepatic clearance of the therapeutic nanoparticles. A secondary benefit is liver toxicity reduction, a common risk with both engineered nanoparticles and viral vectors used to deliver nucleic acid-based therapies.

Preclinical proof-of-concept studies have been conducted to show that payload-free liposomes can be used to increase exposure of nucleic acids in target tissues by temporarily reducing unwanted hepatic clearance [43,44,45]. In these studies, nanoprimers were engineered to be rapidly phagocytosed by Kupffer Cells (KCs) and Liver Sinusoidal Endothelial Cells (LSECs) after intravenous administration. LSECs are scavenger cells with a diameter of 7–9 µm capable of internalizing particles up to 0.23 µm, while KCs are resident liver macrophages with a diameter of 10–15 µm that can take up larger particles than KCs [46,47].

Germain et al. [43] describe the physicochemical attributes claimed to be optimal for liver accumulation. They argue that nanoprimer particles need to be larger than the fenestration size of the space of Disse to be preferentially cleared by KCs and not by hepatocytes, and smaller than 200 nm to avoid spleen accumulation. They also claim that that the charge should be neither positive, a known source of toxicity, nor too negative, to avoid macrophage internalization. More details on different nanoparticle formulations and liver interactions were recently summarized by Zhang et al. [48].

The study by Germain et al. [43] showed that nanoprimers could enhance the anti-tumor efficacy of irinotecan-loaded liposomes (with a diameter of 200 nm) for the treatment of colon cancer. Results from this study showed significantly enhanced biodistribution after application of the nanoprimer. Interestingly, an enhanced availability of the liposomes in the blood resulted in a longer liposome persistence in the head as well. In addition to the increased biodistribution to the head, application of nanoprimers showed a long-lasting effect on the biodistribution which was clearly visible even after a 24 h separation between the intravenous injection of the nanoprimer and intravenous infusion of the therapeutic nanoparticles.

Saunders et al. [42] report a preclinical study in mice where LNPs nanoprimers were used to increase levels of two types of LNP-encapsulated nucleic acid in systemic circulation: Human erythropoietin (hEPO) mRNA and Factor VII (FVII) siRNA. Intravenous nanoprimer pre-dosing 1 h prior to the intravenous administration of the loaded LNPs achieved the desired effects, in this case by increasing hEPO expression and by decreasing FVII expression by 49% (blood concentrations). In the same paper a prior liver panel 24 h after the last of three injections of nanoprimers (administered alone) showed no evidence of toxicity as measured by aspartate aminotransferase (AST), alanine aminotransferase (ALT), albumin, and total protein levels. Furthermore, nanoprimer uptake by liver cells was shown to “reduce KC’s and LSEC’s clearance activity without impacting hepatocyte uptake activity” [Ibid.], thereby suggesting that the vital role of hepatocyte in metabolism, detoxification and protein synthesis had been preserved.

In summary preclinical studies in oncology show that the concept of “nanopriming” is a promising one to enhance the PK of drug-carrying nanoparticles. However, translational implementation is rendered complex by the need to tailor nanoprimer size to organ variations between species. As for viral vectors, it remains unclear to what extent a nanoprimer would be effective at transiently blocking their metabolism. The diameter of Adeno-Associated viruses ranges from 20 to 29 nm [49]. Thus, they may evade the blockading effect of KCs and LSECs by “slipping though” the fenestration of the space of Disse and undergo transcytosis through hepatocytes. Furthermore, AAVs are reported to bind to KCs and LSECs via receptor A and to do so independently of clotting protein Factor X [45]. Whether this binding is sufficient to boost the PK of AAVs remains to be established. Viral vectors would have to bind to KCs and LSEC via receptor A before they slip through the space of Disse. Proof of concept experiments with viral vectors may need to be postponed until pioneers in the nanoprimer field have de-risked the concept. So far biotechnological engineering of the capsid proteins of AAV is another way to obtain enhanced target specificity and less liver clearance, as shown recently [50].

Initial proof-of-concept experiment using nanoprimers should focus on enhancing the PK of large nanoparticles (larger than the space of Disse) to determine if increased brain exposure and half-life can be achieved along with reduced liver toxicity.

### 2.3. Exosomes

Exosomes are cell-derived vesicles. They are actively secreted by most cell types and have a diameter of 40–100 nm [51]. They are formed in the endolysosomal pathway by inward budding of the membrane of the multivesicular body (MVB). Exosomes are subsequently released from the cell after fusion of the MVB with the cell membrane. Exosomes contain various cargos such as mRNA, non-coding RNAs, microRNA as well as cytoplasmic and membrane proteins [52]. Their physiological and pathophysiological role in CNS diseases has been the subject of intensive studies since their discovery more than 30 years ago. [51]. The fact that exosomes are part of the cell-to-cell communication machinery where they fulfill this function by carrying nucleic acids from one cell to another logically gave rise to the idea of developing exosomes and/or other extracellular vesicle as delivery vectors for RNA- and gene therapies. As a drug delivery system, exosomes are an attractive alternative to viral vectors and other nanoparticles. The fact that they are natural particles provides them with several advantages over AAV as well as synthetic nanoparticles (e.g., LNPs). First, they have a low likelihood of stimulating the immune response as their immunogenicity is lower than that of other delivery vehicles such as viruses or liposomes [53], making them more suitable for repeat dosing. Viral vectors such as AAV have a high propensity to stimulate the immune system resulting in a limited number of doses that can be given to a patient. Similarly, exosomes do not elicit any toxicities due to the natural lipid content of their membranes, a problem often seen with synthetic nanoparticles. Furthermore, the likelihood of being cleared from the circulation by the liver and the reticuloendothelial system (RES) or the immune system is lower. Depending on the therapeutic indication—and thus the target organ that needs to be reached—exosomes can provide a lot of flexibility: As they have an intrinsic targeting ability for different target tissues, exosomes with different targeting profiles can be generated by changing the producing cell line. In addition, exosomes can be genetically engineered to further refine their targeting towards a target organ such as the brain. According to the literature, brain targeting has been accomplished by attachment of peptide-based targeting ligands such as RVG29, g7 or RGD-Dyk. A recent study showed that conjugation of rabies virus glycoprotein-derived peptide (RVG-29) to the exosome surface enhanced brain uptake of the exosomes three-fold [53]. However, none of the peptide-based targeting ligand has been clinically validated yet.

Thus, exosomes have gained much attention as drug delivery vehicles for the treatment of CNS diseases and gene therapy approaches in recent years. Below are just some examples of preclinical studies. Exosomes carrying micro-RNA miR-133b reduced the infarcted area and improved neurological deficits in a middle cerebral aortic occlusion (MCAO) rat model of brain ischemia [54]. A study by Matthew Wood’s group showed that conjugation of the rabies virus glycoprotein (RVG-29) peptide to the exosome surface enhanced brain uptake of the exosomes two-fold. BACE1 and Aβ levels were significantly reduced in mouse cortex after intravenous application of RVG-29-coated exosomes loaded with siRNA against BACE1 [55]. The same group confirmed efficient brain delivery of RVG-29-coated exosomes in a Parkinson disease mouse model. A significant reduction in α-synuclein mRNA and protein levels was also observed in several brain regions after injection of RVG-29-coated exosomes loaded with siRNA against α-synuclein [56], but exosome treatments are increasingly finding their way into the clinics as well. The first clinical studies using autologous exosome-based therapies demonstrated good safety and tolerability [57,58]. A recent (January 2022) search in clinicaltrials.gov found 116 clinical studies for “exosomes”. While most of these studies use exosomes for diagnostic purposes, 35 studies are pursuing therapeutic interventions using exosomes as delivery vehicles. However, only three of these studies try to treat CNS diseases. The indications mentioned for these studies are stroke (NCT03384433, Ph1/2), depression, anxiety, and dementia (NCT04202770), phase not disclosed) and AD (NCT04388982, Ph1/2). Interestingly, exosomes in study NCT04202770 were applied using focused ultrasound (FUS), thus avoiding BBB transport but relying on transient local opening of the BBB. Currently, another clinical study is investigating the safety and efficacy of allogenic mesenchymal stem cell-derived exosomes in AD patients (NCT04388982).

As a result, various companies (see Table 3) have come into business which develop exosomes as therapeutics for various diseases, some of them already in clinical phases.

Recently the field has moved another step ahead with big pharma companies entering the picture [59]. The first business deals between exosome companies and big pharma companies were announced as early as 2017 when Boehringer Ingelheim and Evox Therapeutics announced a research collaboration on exosome-mediated RNA delivery in Boehringer’s therapeutic areas of interest. Over the last two years, additional business deals between small companies developing exosomes as delivery vehicles for therapeutics and big pharma companies have been disclosed, which demonstrate big pharma’s increasing interest in this technology. Among the biggest of them are Lilly’s deal with Evox Therapeutics on CNS-targeting exosomes for five undisclosed targets which could give up $1.2 billion in milestone payments and Takeda’s $900 million deal with Carmine Therapeutics on the development of gene therapies against two undisclosed rare disease targets. This is illustrated in Table 4.

Autologous exosome-based therapies were well tolerated in first clinical cancer studies [58,69]. Still, some issues remain that hinder a broader clinical application of exosomes in CNS disease therapies. First, different functions of exosomes in health and disease have been identified and are not completely understood. These may even be opposing, as exosomes on the one hand mediate tumor prevention, but on the other hand deliver tumor-associated proteins. Selection of the right cell types as starting material for exosome production is important to avoid possible side effects given the varying intrinsic contents of exosomes because of their cellular origin. Mesenchymal stem cells seem to be a suitable source of exosomes, as they have been used in the three clinical studies. In addition, it is still unclear by which mechanism exosomes cross the BBB. Exosomes mostly interact with the cell membrane via membrane fusion, and subsequently deliver their cargo into the cell. Recent studies suggest that exosomes can take different routes through the cell. A routing towards late-endosomes/lysosomes from which they can be released via transcytosis has been described in zebrafish macrophages. Furthermore, they also seem to cross an in vitro BBB via transcellular migration, but only under stroke-like conditions. However, the therapeutic relevance of this route is questionable since disruption of the BBB in stroke occurs after the therapeutic window for treatment initiation [70]. The BBB also is a formidable barrier in other brain diseases such as glioma [71].

A focused optimization of exosomes as delivery systems will only be possible with a deeper knowledge of these processes. In addition to these biological questions, there are technical issues to be solved. Loading exosomes with nucleic acids requires optimization. There are different methods of bringing a cargo into exosomes. Several physical techniques have been reported to enable uptake of cargos into exosomes [72]. Such techniques include sonication, electroporation, and surfactant treatment. However, loading efficiencies of maximally 25% have been reported for most techniques. The loading of nucleic acids can also be achieved by transfecting the producing cells which then overexpress the nucleic acid. The produced exosomes will then contain the expressed nucleic acid. Consequently, a high number of exosomes must be delivered to achieve therapeutic concentration of the drug in the target tissue. Especially for CNS diseases, an improved brain targeting resulting in higher brain uptake would be desired. Although exosomes have an intrinsic propensity to enter the brain, a recent study in mice suggests that only about 0.05% of an injected dose of non-targeted exosomes crosses the BBB [73]. Surface modification of exosomes can increase their brain-targeting efficiency. A recent study showed that conjugation of RVG-29 peptide to the exosome surface enhanced brain uptake of the exosomes, but only by three-fold. The identification of a more efficient targeting ligand will be required to enable a stronger increase in brain uptake. Lastly, production of exosomes in sufficient amounts to support clinical studies is another issue that needs to be solved. Leukapheresis and subsequent ex vivo generation of autologous exosomes has shown to provide sufficient material for clinical studies. Recent technical developments using dynamic 3D cultures suggest a path forward for non-autologous exosomes as delivery systems, as this resulted in a 100-fold increase in yield over the use of 2D cultures. Exosome companies also claim that their proprietary production platforms are scalable and suitable for large scale applications. The fact that some of these are in clinical studies seems to underline this fact.

### 2.4. Exo-AAVs: Exosomes as Capsid Carriers

Although exosomes move forward slowly towards the market, it is still early days for this technology. Solutions for the issues mentioned are needed for exosomes to progress further as delivery vehicles, especially for CNS therapeutics, but since the field is growing very quickly, additional knowledge to these issues should emerge soon and support the progression of exosomes into the clinic and to the market. Already, first approaches are being developed that broaden the use of exosomes by transporting cargos beyond nucleic acids. An interesting novel application of exosomes developed in recent years is exo-AAVs (also named “vexosomes” or “ev-AAV” (extracellular vesicle-associated AAV), where AAVs associate with exosomes. In this approach, exosomes generated by HEK293T cells transfected with AAV plasmids for AAV1 and AAV2 and isolated from culture medium were found to contain AAVs either associated with the exosomes or even within the exosomes. Approximately 12 and 9% of isolated exosomes contained AAV1 and AAV2 capsids, respectively and the average number of capsids per exosome was 8.2 and 1.2 for AAV1 and AAV2 [74].

In vitro studies transfecting U87 cells, either with AAV1 or AAV1 vexosomes after pre-incubation with an anti-AAV1 neutralizing antibody showed that AAV1 vexosomes yielded a more than four-fold higher transduction efficiency than AAV1. AAV1- and AAV2-vexosomes also showed higher transduction rates compared to free AAV capsids even in the absence of neutralizing antibodies [74]. In vitro studies then showed that ev-AAV containing AAV9 capsids were up to 23 times more resistant to neutralizing antibodies in transducing HeLa cells than AAV9 capsids alone. Furthermore, intravenous injection of a neutralizing dose of immunoglobulin (IVIg) from multiple donors in mice followed by application of either AAV9 or ev-AAV9 showed an increased transduction efficiency in both liver and head as demonstrated by a 5-7-fold higher luciferase signal. This demonstrated that ev-AAV9 are less sensitive to IVIg than free AAV9. Interestingly, decorating the EVs with RVG as a brain targeting ligand further increased delivery of ev-AAV9 to the brain by 3-4-fold. Interestingly, transduction in liver and heart was strongly reduced with RVG-ev-AAV9 compared to untargeted ev-AAV9 [75]. Another in vivo study showed that intravenous application of exo-AAV9 in mice not only resulted in a similar distribution of the introduced EGFP signal throughout the whole brain compared to free AAV9, but they also showed comparable cellular tropisms in the brain. At the same time, the results showed a significantly higher transduction efficiency of ev-AAV9 compared to free AAV9 in cortex and striatum but not in the hippocampus [76]. However, ev-AAVs show potential beyond the brain. A recent study showed increased efficacy of ev-AAV (here called AAVExo) in a mouse model of lung cancer, where AAVExo demonstrated a significantly higher delivery to the xenografts compared to free AAV [77].

The exo-AAV approach may offer several advantages. Although AAVs are widely used as delivery vehicles for gene therapy approaches, a low likelihood of stimulating an immune response is one of the biggest advantages of exosomes over AAV, especially for repeated dosing. While methods to avoid an immune response after AAV application such as empty capsid decoys [74], balloon catheters and saline flush or removal of neutralizing antibodies from the blood by plasmapheresis [75] have been developed, these methods also have their limitations. Technologies such as plasmapheresis are elaborate technologies which place a heavy burden on the patient and can only be repeated a few times. Protecting the capsids from neutralizing antibodies seems indeed possible with ev-AAVs, as in vitro data suggest. The biodistribution of exosomes shows a majority accumulating in the liver. This is a concern because recent literature [50] highlights the need to keep capsids from entering the liver at a high percentage as this can trigger responses such as liver toxicity. Whether encapsulation of capsids into exosomes can increase safety needs to be demonstrated; at least the data with RVG-ev-AAV9 suggest the possibility to reduce delivery to the liver. This may also be helpful as a large fraction of AAV capsids is sequestered in the liver upon intravenous dosing. On the other hand, the intrinsic targeting property of exosomes may help direct an increased fraction of the ev-AAVs to the target organ, especially the brain, as exosomes possess an intrinsic property to cross the BBB. The in vivo results with RVG-ev-AAV9 suggest that an increased delivery into the brain can be achieved.

While this approach has some intriguing implications, it is still at the early stage. Several points need clarification to objectively assess the full measure of its potential and limitations. Currently, information on exo-AAV in the literature remains limited. Second, only about 10% of the exosomes contain capsids (which is in line with low loading efficiencies for exosomes). Conversely, exosomes can contain up to eight capsids. This means that controls should be put in place to ascertain that the claimed higher transduction of brain cells is indeed based on a higher number of capsids/viruses in the brain. None of the studies so far has confirmed this claim. This is a concern because the mechanism by which exosomes cross the BBB remains unclear. Lastly, optimization of the isolation method to increase the degree exosome loading has not been reported to date.

## 3. Device-Enabled Drug Delivery

Apart from drug delivery technologies such as receptor-mediated transport (RMT) or intranasal delivery (IN), there are device-enabled drug delivery technologies which allow local delivery to specific regions of brain parenchyma or regions throughout the brain or spinal cord via cerebrospinal fluid (CSF). These technologies are either invasive, such as intracerebroventricular (ICV), intra-cisterna magna (ICM) or intrathecal (IT) injection, or non-invasive such as focused ultrasound (FUS) for BBB disruption. ICM injection is more convenient than ICV application in rodent studies. For safety reasons, however, ICM cannot be used routinely in clinical trials. Both routes of administration provide efficient access to the subarachnoidal space.

### 3.1. Focused Ultrasound (FUS)

Multiple approaches have been used to disrupt the BBB using focused ultrasound. Disruption of the BBB with focused ultrasound can be accomplished with microbubble injections using implanted transducers or non-invasive phased-array transducers. The biological effects and associated safety profile of the disruption procedure significantly depend on the magnitude of applied ultrasound energy, typically measured as peak negative pressure. The concomitant systemic circulation of injected microbubbles allows a reduction of the peak negative pressure (and ultrasonic energy) needed for BBB disruption. Sizeable peak negative pressure results in irreversible BBB damage while reduced peak negative pressure can avoid irreversible BBB damage. A recent screen in clincialtrials.gov as well as recent literature [78] identified 17 clinical trials using FUS technology. Table 5 provides a summary of the safety assessment period for several clinical trials that utilize microbubbles to disrupt the BBB.

The safety of disrupting the BBB using microbubbles has been controversial [5,79,80]. Vikram Patel, former deputy director of the Division of Applied Regulatory Science at the Center for Drug Evaluation and Research at United States Food and Drug Administration (FDA) commented specifically on the safety aspects of closing the BBB “as soon as possible to limit exposure of the brain to chemicals or toxins other than the intended therapeutic compounds”. Kovacs et al. [81] provided evidence that even relatively low ultrasonic energies used with Optison^®^ microbubbles induced immediate expression of damage associated molecular patterns (DAMP), heat shock protein 70 and other cytokines associated with a sterile inflammatory response. These patterns are also found in traumatic brain injury. Recently Schregel et al. [82] delivered an Optison^®^ microbubble dose in combination with the same peak negative pressure used in the experiment by Kovacs et al. [83] to intentionally induce focal lesions resulting in experimental autoimmune encephalomyelitis (EAE) as a disease model of multiple sclerosis in mice.

Accumulation of blood factors in the brain has been implicated in neuroinflammation and neurodegeneration in the CNS. Thrombin activates NF-κB signaling in microglia to promote oxidative stress and activates pro-inflammatory response in microglia, contributing to neuronal cell death [83]. Fibrinogen regulates inflammation in the CNS and contributes to neurodegenerative progression [84]. Fibrinogen accumulation in oligodendrites leads to myelin loss and axon degeneration, leading to cell death [85]. Complement system acts as a key regulator in glial phagocytosis and contributes to cytokine production and inflammatory response in the brain [86,87]. This means that FUS parameters would need to be tightly controlled to avoid the crossing of the BBB by blood coagulation factors.

FUS technology, as currently designed, cannot be used to increase BBB permeability efficiently and transiently over wide areas of the brain. Accordingly, there have been no clinical studies that evaluate disruption of large volumes of the brain while co-administering a therapeutic drug yet. Due to the limited focal area of the ultrasound beams, disruption of a large area of the brain would either require a highly multiplexed disruption algorithm to target multiple cortical volumes at once or a steerable ultrasound beam to achieve widespread coverage (or both). The largest volume of disruption evaluated to date is approximately 24 cm^3^ [88], which would require nearly 60 “large volume” treatment sessions of considerable duration to treat the entire brain volume. As such, the approach would not be practical.

Furthermore, co-localization and synchronization of the ultrasound field, microbubbles and therapeutic agents are a particularly challenging obstacle to optimizing drug exposure at the target tissue of interest. The timing of delivery for optimal concentration of the microbubble and therapeutic agent with the ultrasound field at the small target site is critical to achieve consistent passage of the therapeutic agent to the target tissue when BBB disruption is minimally transient. Microbubbles travel through the lungs before entering the arteries within the brain, and a loss in the concentration of microbubbles in the lungs is to be expected [89]. Since the degree of BBB disruption is dependent on the concentration of microbubbles [90], the magnitude of disruption during a single pulse sequence is necessarily very dynamic.

In addition, infrastructure costs and requirements to properly perform the BBB disruption procedure are high [80] and limit access for patient treatments. The cost for a single InSightec Exablate^®^ 4000 system is estimated at $2,000,000 [91] and a single ablation treatment for essential tremor is estimated at $23,500 [92]. If these costs are similar for a MRgFUS system for BBB disruption, there will be significant reimbursement hurdles utilizing this technology to enhance drug distribution.

Another important point to consider is the fact that microbubbles are currently classified as drugs for use as contrast agents to be used in conjunction with diagnostic ultrasound equipment. A different intended use of microbubbles to mechanically disrupt the blood brain barrier is likely to fit the definition of a medical device, which would require a change in classification from drug to device.

Even in the best-case scenario, i.e., “one time” drug delivery to limited regions of the brain (a few cubic millimeters), several technical challenges remain to be solved. This is compounded by safety concerns, commercially available microbubbles originally approved as contrasting agents (i.e., a different intended use) and complex regulatory hurdles on which experts in the field have yet to provide clear pathway to approval.

Despite years of feasibility experimentation reported in numerous publications and conference presentations, FUS applications for widely disrupting the BBB cannot be counted as a scalable platform for commercial development in the foreseeable future. In this context, when we consider more challenging scenarios, e.g., large neuroscience markets where drug exposure is required over large cortical volumes, or even more daunting, where repeated doses are required over large cortical volumes, the prospects for a breakthrough with current FUS technology are very low. However, several promising innovations, such as the use of Rapid Short Pulse (RaSP) ultrasound, may become more viable technologies for future clinical studies.

### 3.2. Intra CSF Delivery

In a recent review paper [93], we highlighted that there are two schools of thought on the delivery of large proteins to deep brain tissues via the cerebrospinal fluid (CSF). The early school posits that the rate-limiting role of molecular size on protein diffusion through the interstitial space limits the utility of intra-CSF delivery to the treatment of brain tissues that are either located within, or in contact with, the CSF ventricular/ meningeal system as in the case of leptomeningeal metastases. A more recent school has gained recognition following advances in ex vivo and in vivo imaging that revealed the existence of a CSF “microcirculation” system within brain tissues, at times, referred to as the “glymphatic system” [94] As a result, the CSF circulation that was first thought to be useful only to reach tissues in the immediate vicinity of the CSF circulatory system, is increasingly viewed as a pathway for a more rapid intake of therapeutics into deep brain tissues.

Under this new paradigm, deep penetration of the brain by large molecules is achievable via perivascular pathways as demonstrated in primates [95]. The existence of deep penetrating perivascular pathways also has far-reaching implications on cross-species translation and on the effect of molecular weight of CSF solutes on rate of transport within the parenchyma. Such pathways allow micro-convective transport that scales with brain size. Under the intra-parenchymal CSF diffusion paradigm, CSF enabled-drug transport was widely regarded not to be scalable to higher order species. Diffusion was seen as limiting penetration distance and as presenting a major hindrance to the penetration of therapeutics with high molecular weight. However, over the past two decades there have been multiple clinical studies employing ICV injection. First indications for efficacy have been seen in these studies [96,97] suggesting therapeutic concentrations of the drug in the parenchyma.

The implications for drug delivery via CSF microcirculation are far reaching, as shown in a widely cited paper by Yadav et al. [95]. They observed widespread distribution of an anti-BACE1 antibody and of a control IgG antibody after 6 weeks of ICV infusion in primates. Continuous ICV delivery of an anti-BACE1 antibody achieved a steady-state concentration in the CSF within 4 days. Antibody distribution was near uniform across the brain parenchyma after 6 weeks, ranging from 20 to 40 nM, which led to a robust and sustained reduction (~70%) of CSF amyloid-βx-40 peptides. A rat study by Kouzehgarani et al. [98] also demonstrated the suitability of CSF microcirculation for drug delivery. They showed that an antibody penetrates the brain parenchyma starting approximately 30 min after a single intra Cisterna Magna (ICM) injection and continues to do so even 4 h after injection. Even after 24 h the antibody is still present in the tissue.

In contrast, no tissue penetration was observed 4 h after intravenous injection, even with a 30-fold higher dose than in the ICM study. The penetration extended into subcortical regions including the hippocampus. Similarly, two studies performed in Tg2576 mice demonstrated a therapeutic effect of chronically ICV-infused antibodies after 5 and 2 weeks of infusion, respectively [99,100]. This pioneering research has shown that the intra-CSF delivery of large protein therapeutics can achieve widespread distribution and target engagement in deep brain tissues. This indicates that intra-CSF administration can be an attractive option to reach and engage targets in brain tissues when (a) the BBB is poorly permeable to the therapeutic such as in the case of vectorized nucleic acid therapeutics, degradomers or biopharmaceuticals, (b) widespread areas of the brain need to be treated which is not achievable with local delivery with modalities such as FUS or intra-parenchymal injections/infusions, (c) systemic delivery would either lead to unacceptable off-target toxicity, or (d) lead to the degradation or rapid clearance of the therapeutic, and (e) there is no evidence of brain toxicity. Finally, the severity of the condition and treatment benefits may justify an invasive route of administration in the patient. In such cases, intra-CSF administration can provide a convenient short cut to collect proof of concept data on the efficacy of a therapeutic.

Several studies have been published showing that maintaining a steady-state drug concentration in the SAS is a necessary condition to reach deep areas of the brain [95,101,102]. This is to counteract the dilutive effect of rapid CSF turnover (ranging from 13 times a day in mice to 4 times a day in humans). Thus, determining a suitable intra-CSF dosing regimen requires modeling, CSF sampling studies or a combination of the two.

The above concept is well illustrated in a study by Fleischhack et al. [103] comparing bolus ICV administration with continuous intravenous (CIV) administration of chemotherapy agent Etoposide in patients with metastatic medulloblastoma. Five daily ICV bolus doses (0.5 mg per day) via an indwelling subcutaneous reservoir achieved more than a 100-fold peak exposure compared to intravenous infusion. CSF clearance caused each peak to be followed by a steep trough, however. Repeated intraventricular etoposide administration was only minimally toxic, was well tolerated, and steady state ICV exposure exceeding that achieved with continuous intravenous infusion was suggested as a follow-up study.

#### Route of Intra-CSF Administration for Deep Brain Penetration

Intra CSF administration for delivery to the brain can be achieved via intrathecal (IT), intra-cerebroventricular (ICV) or intra-cisterna magna (ICM) infusion. As seen in Table 1 all ongoing clinical studies in the indications that we screened for used the IT or the ICM route for drug application. However, one clinical trial is currently in Ph2a/b (NCT04153175) in which sodium valproate is administered intra-cerebroventricularly to treat refractory epilepsy [104].

Until recently, the IT route was preferred over ICV route whenever possible due to the risk of infection from long-term use of ICV. For the treatment of deep regions of the brain, excluding ICV delivery comes at a cost. With CSF “near stagnant” in the lumbar area, IT delivery requires pumping a higher initial dose to reach the subarachnoid space (SAS) at therapeutic levels, a precondition to sustained penetration via the microcirculation system. In contrast, ICV leverages brain physiology by infusing the drug in proximity to the site of CSF production, thereby allowing the drug to go with the outward flow of the CSF in the direction of the SAS. This is illustrated by a study published by Vuillemenot et al. [105] describing TPP1 enzyme replacement therapy to treat CLN2, an ultra-rare and rapidly progressing brain disorder that affects an estimated 20 children born in the United States each year. When comparing IT and ICV delivery of TPP1 in primates treated with a single infusion, they found that although CSF and plasma PK profiles were equivalent between ICV or IT infusion, ICV infusion achieved increased TTP1 exposure in all areas of the brain, particularly in the striatum and the thalamus.

The above case studies in both rodents and primates confirm the scalability of ICV delivery and, more generally, the viability of harnessing CSF microcirculation across species with major difference in brain size, a notion that continues to be met with skepticism in some neuroscience circles. However, the pharmaceutical industry is beginning to take note of the promise of ICV for intra-CSF delivery, as exemplified by Genentech in the aforementioned anti-BACE-1 study, which was conducted in collaboration with Medtronic.

IT delivery is well suited for the delivery of lipophilic drugs to the spinal cord area, with diminishing utility for reaching the cerebral SAS due to their rapid absorption in the spinal cord SAS. Moreover, the low rate of CSF circulation in the lumbar area further increases therapeutic concentrations within the confined of the spinal SAS. The situation is less clear cut in the case of therapeutic proteins. While therapeutic proteins do not readily bind to spinal tissues, their distribution beyond spinal CSF remains hindered by the slow CSF circulation in the lumbar area. This hurdle can be overcome by infusing the drug with an implanted pump to steadily replace the drug fraction that is cleared from the cervical area. In fact, IT application of morphine for pain relief was described as early as 1979 [106]. More recently Prialt^®^ has been added to that list, which is the only FDA-approved non-opioid medication for the treatment of chronic pain [107]. Furthermore, intrathecal baclofen is approved by the FDA for the treatment of spasticity [108].

To better understand the role of intra-CSF delivery in the treatment of spinal cord injury (SCI) a search of ongoing clinical trials was conducted on the “SCI clinical trial” website [109]. The first screening excluded non-pharmaceutical modalities as well as chronic management of co-morbidities. The search results revealed that four of nine SCI studies utilized IT delivery as shown in Table 6. When focusing on those SCI trials where the mode of action for neural regeneration was either repulsive guidance molecule receptor A (RGMA) pathway inhibition (AbbVie’s ABT-555 and Mitsubishi’s MT-3921) or NGR-1 pathway inhibition (ReNetX Bio’s AXER-204 and Novartis’s NG-101-ATI355), two of these studies utilized intravenous delivery. Published literature indicated that intravenous delivery for MT-3139 (anti-RGMA from Mitsubishi) was chosen out of clinical convenience after successful POC in primates using IT delivery [110,111,112,113,114]. In contrast, AXER-204 and NG-101-ATI355 were dosed in humans via the IT route after successful preclinical studies in primates.

### 3.3. Convection Enhanced Delivery

The Blood-Brain-Tumor Barrier (BBTB) in GBM tumors can be highly heterogeneous, with permeability to protein therapeutics ranging from high at the tumor core of high grade GBM to low, or extremely low, in unresectable regions such as tumor margins or sparsely infiltrated areas distal from the core. Furthermore, there is mounting evidence from preclinical and clinical studies that BBTB heterogeneity significantly limits efficacy following systemic delivery [115].

To evaluate this hypothesis, a preclinical study in mice was conducted to explore the benefits of administering two antibody-drug conjugates, Depatuxizumab mafodin (ABT-414) and Serclutamab talirine (ABBV-321), via convection enhanced delivery (CED) as an alternative to systemic delivery [116]. Efficacy was evaluated by using an intracranially implanted EGFRviii-amplified patient-derived xenograft (PDX) refractory to treatment via intraperitoneal (IP) delivery (implying BBB impermeability). Four consecutive doses of ABT-414 administered with CED led to a 5-fold increase in mouse longevity compared to weekly systemic administration. It was concluded that ABT-414 is well tolerated as infusion was only associated with modest elevation in glial fibrillary acidic protein (GFAP) without loss of NeuN staining or increased infiltration of CD68-positive cells and resulted in extended survival in orthotopic GBM PDXs. Similarly, a single dose of ABBV-321 administered with CED led to a significant increase in mouse longevity compared to weekly systemic administration. With intra-tumoral administration, two out of three mice were still alive after 300 days vs. a median survival of <60 days with the systemically dosed negative controls. However, ABBV-321 had a much narrower therapeutic window when delivered by CED. More importantly, this study showed that CED is a promising method to enhance delivery across the BBTB.

Publications by Hadaczek et al. [117] and Johnston et al. [118] also describe CED protocols to deliver AAV2-GDNF, AAV2-TK in the striatum and putamen regions of monkeys, resulting in prolonged increases in dopaminergic neural activity and associated locomotor activity.

A necessary condition for CED to be an option is when the regions of the brain requiring drug exposure are precisely located and of relatively small size. Examples of conditions meeting these criteria are clinical study NCT04120493 for early-stage HD, clinical study NCT04167540 for early or late-stage PD, or clinical study NCT01621581 for advanced PD.

## 4. Conclusions and Outlook

Brain delivery technologies, non-invasive, invasive, and device-mediated, are in different states of maturity. Each of these has specific properties that may be useful for different indications, e.g., local vs global delivery. Table 7 summarizes the properties of delivery technologies sharing the function of enhancing the brain biodistribution of therapeutics ranging from small molecules, degraders, biologics to nucleic acids. The null hypothesis assumes that a technology works as intended until proven otherwise.

Exosomes are currently a hot topic, and various companies have started to invest heavily into this technology. However, despite considerable progress made over the last 10 years, there are still many unresolved issues. Still, exo-AAVs represent an exciting novel development. Although this approach is in an even earlier stage it may present an intriguing opportunity to broaden the use of AAVs in indications that require repeated dosing. To our knowledge nanoprimers are the first technology that tries to concomitantly modulate both the biodistribution and the PK of nanoparticles. While this improves the chances for a successful development for peripheral indications, it does not resolve the issue of opsonization (corona formation) and the resulting loss of brain targeting efficacy. Although the first published results look quite promising, the available literature on nanoprimers remains scarce. Future studies will show if this technology can live up to its promise. In addition, the field is currently lacking a clinically validated targeting ligand.

Focused ultrasound (FUS) technologies have limited preclinical utility in niche areas. A review performed on FUS shows that the technology does not fully live up to the expectations set by its advocates in academic circles as a scalable drug delivery technology. The current embodiments of the technology are not suited to treat large areas of the brain and suffer from safety and regulatory issues that make it difficult to implement beyond phase 1.

Device-based methods to circumvent the BBB, such as convection enhanced delivery (CED) or intra-cerebroventricular delivery (ICV), show more promise preclinically and clinically. There are scalable solutions from the clinic and to commercial launch. The Renishaw NeuroInfuse^®^ system is suitable for both acute (single delivery) and chronic repeat delivery paradigms, whereas other CED equipment suppliers only have delivery solutions for acute delivery paradigms. Cerebral Therapeutic’s ICVRx^®^ Infusion System is a fully implanted delivery system that consists of a dual lumen port and ICV catheter, enabling ICV infusion as well as aspiration of ventricular CSF for biomarker analysis. The ICVRx^®^ is attached to either a port or refillable pump to allow intermittent or continuous delivery, respectively. The fully implanted design improves the safety profile over designs requiring an external pump interface.

A recent study in rodents has shown that a humanized antibody administered via CSF penetrated the entire brain at a rate and depth that has surprised many subject matter experts. The circulatory pathway leading to this rapid tissue exposure is described in a 2021 review paper [93]. Benchmarking shows that some companies use device-based modalities for the delivery of nucleic acid in clinical studies. While device-based modalities currently would clearly be impractical for large primary indications, the authors of this paper recommend considering implantable ICV to achieve rapid results in niche markets, particularly those involving acute morbidities with rapid progression. ICV delivery should also be considered for target validation studies, even if the eventual preferred mode of delivery is systemic. To this end, it would be interesting to validate the corona mimicry approach for its ability to maintain the brain targeting capability of nanoparticles.

## Figures and Tables

**Table 1:** Approved gene therapy-based drugs for the treatment of CNS diseases.

Approved	Drug	Indication	Delivery System & Route of Administration	Therapeutic
2016	Spinraza	Spinal Muscular Atrophy	(intrathecal)	Antisense oligonucleotide against SMN2
	Luxturna	Inherited Blind Diseases	Adeno-associated virus Type 2 (subretinal injection)	Retinal pigment epithelium-specific 65 kDa protein
	Brineura	Batten’s Disease	(intra-cerebroventricular)	Recombinant TPP1
2019	Zolgensma	Spinal Muscular Atrophy	Adeno-associated Virus Type 9 (intravenous)	SMN1
2021	Delytact	Malignant Glioma	Herpes Simplex Virus Type 1 (intra-tumor)	(oncolytic)

**Table 2:** On-going Gene Therapy Trials and Routes of Administration by Disease Indication.

Route of Administration (No. of Studies)	AD	PD	FTD	HD	Gaucher	ALS
Systemic (0)	0	0	0	0	0	0
Intra-Cisterna Magna (5)	NCT03634007 Ph.1/2 2018–2023 (est) NRP	NCT04127578 Ph.1/2 2020–2028 (est) NRP	NCT04747431 Ph.1/2 2021–2027 (est) NRP	0	NCT04411654 Ph.1/2 2021–2028 (est) NRP	0
			NCT04408625 Ph.1/2 2020–2027 (est) NRP			
Intrathecal (4)	NCT03186989 Ph.1/2 2017–2022 NRP	NCT03976349 Ph.1 2019–2023 (est) NRP	0	0	0	NCT04494256 Ph.1/2 2020–2026 (est) NRP
						NCT04856982 Ph.3 2021–2027 (est) NRP
Intra-Parenchymal (7)	0	NCT01621581 Ph. 1 Convection-enhanced delivery 2013–2022 RP [9]	0	NCT04120493 Ph.1/2 MRI-guided infusion 2019–2029 (est) NRP	0	0
		NCT04167540 Ph.1 Bilateral image-guided infusion 2020–2027 (est) NRP				
		NCT3720418 Ph.1/2 Neurosurgical delivery 2018–2022 RP [10]				
		NCT01856439 Ph.1/2 Bilateral injection 2011–2022 RP [11]				
		NCT03065192 Ph.1 Neurosurgical infusion 2017–2021 RP [12]				
		NCT03562494 Ph.2 Brain infusion 2018–2023 (est) RP [9]				

NRP = no results posted on www.clinicaltrials.gov (accessed on 16 January 2023); RP = results published; est = estimated study completion date.

**Table 3:** Companies Developing Exosome Technologies.

Company	Website (Accessed on 22 March 2023)	Technology	Status
Aegle Therapeutics (Woburn, MA, USA)	www.aegletherapeutics.com	Production of therapeutic-grade extracellular vesicles from bone marrow derived mesenchymal stem cells (MSCs)	Ph1/2a (Imm)
Anjarium Biosciences (Schlieren, Switzerland)	www.anjarium.com	proprietary Hybridosome^®^ delivery technology for non-viral gene therapy	Not disclosed
Aruna Biomedical (Athens, GA, USA)	www.arunabio.com/	Proprietary neural exosomes AB126 (derived from proprietary non-transformed neural stem cells) able to cross the BBB	Preclinical
Capricor Therapeutics (San Diego, CA, USA)	www.capricor.com	Exosomes from proprietary cardiosphere-derived cells and engineered exosomes	Preclinical
Carmine Therapeutics (Cambridge, MA, USA)	www.carminetherapeutics.com	Red blood cell Extracellular Vesicle (RBCEV) Gene Therapy (REGENT^®^) for the development of next-generation non-viral gene therapies	Not disclosed
Codiak BioScience (Cambridge, MA, USA)	www.codiakbio.com	Proprietary engExTM platform for designing, engineering, and manufacturing novel exosome therapeutics	Ph1, preclinical for NS
Curexsys (Göttingen, Germany)	www.curexsys.com	Induced mesenchymal stem cells (iMSC) derived exosomes isolated via traceless purification of exosomes (TACS)	Ph2
Evox Therapeutics (Oxford, UK)	www.evoxtherapeutics.com/	Modified exosomes targeting the BBB with the RVG peptide	Preclinical
Exopharm (Melbourne, Australia)	www.exopharm.com	Exosomes as delivery systems for RNA, enzymes, or small molecules, with the possibility of surface modification for specific tissue targeting	Preclinical
ILIAS Biologics (Daejeon, Republic of Korea)	www.iliasbio.com	Proprietary EXPLOR^®^ platform for intracellular delivery of large-sized protein therapeutics. BBB-targeted exosomes for CNS diseases	Ph1 (inflamm.), preclinical for NS
Kimera Labs (Miramar, FL, USA)	www.kimeralabs.com	Production of MSC-derived exosomes for cosmetic use and scientific and clinical research	Not disclosed
Reneuron (Bridgend, UK)	www.reneuron.com	modified exosomes for brain targeting, according to their website even to specific brain regions	Ph 2b (stroke)
Vesigen Therapeutics (Cambridge, MA, USA)	www.vesigentx.com	Engineered ARrestin-domain 1 Mediated Microvesicles (ARMMs) as a flexible platform for therapeutic delivery	Not disclosed
Mantra Bio (South San Francisco, CA, USA)	www.mantrabio.com	REVEALTM, an exosome engineering platform that to generate targeted exosome vehicles (TEVs) for various therapeutic areas	Not disclosed
Xollent Biotech (Raleigh, NC, USA)	www.xollentbio.com	A variety of applications for exosomes of different origin in oncology, cardiology and cosmetics	Preclinical

**Table 4:** Business Deals Between Exosome Companies and Big Pharma (from [59], modified).

Companies	Details
Vesigen Therapeutics	Series A round for developing ARRDC1-mediated microvesicles delivering cargos for gene editing, mRNA replacement and RNAi therapeutics ($28.5 million) [60]
Carmine Therapeutics; Takeda	Research agreement using Carmine’s proprietary extracellular vesicles (EVs) for the delivery of Takeda’s gene therapies against two undisclosed targets (Camine eligible for milestone payments up to $900 million) [61]
Curexsys GmbH; Evotec	Partnership combining Evotec’s proprietary induced pluripotent stem cell (iPSC) platform with Curesys’ proprietary exosome isolation technology [62]
Codiak Biosciences; Jazz Pharmaceuticals	Strategic collaboration on the research and development of exosome-based therapies for the treatment of cancer. (Codiak eligible for up to $200 million in milestone payment [63]
Sarepta Therapeutics	Research agreement using Codiak’s engineered exosomes for the delivery of Sarepta’s gene editing, gene therapy and RNA technologies against neuromuscular diseases (Codiak eligible for up-front and license payments of up to $72.5 million) [64]
Evox Therapeutics; Eli Lilly	Research agreement using Evox’s exosomes for the delivery of Lilly’s RNAi and antisense oligonucleotide (ASO) therapies against 5 undisclosed targets in neurological disorders (Evox eligible for milestone payments up to $1.2 billion) [65]
Boehringer Ingelheim	Research collaboration on exosome-mediated RNA delivery in Boehringer’s therapeutic areas of interest [66]
Takeda	Partnership agreement to develop up to five exosome-based therapeutics for the treatment of rare diseases [67]
ReNeuron; undisclosed partner	Research agreement using ReNeuron’s human neural stem cell-derived exosomes to deliver gene-silencing technology of an undisclosed partner [68]

**Table 5:** Clinical Studies Utilizing FUS with Microbubbles to Disrupt the BBB.

Safety Assessment Period	Date Posted	Microbubble Type	Sponsor	Indication	NCT ID
1 day	August 2019–January 2027	Definity^®^ microbubbles	Neurological Assoc., BrainSonix Corp, Sherman Oaks, CA, USA	Low Grade Glioma	NCT04063514
3 days	October 2019–July 2021	Definity^®^ microbubbles	Elisa Konofagou^®^, Columbia University, NY, USA	Alzheimer’s Disease	NCT04118764
90 days	December 2016–June 2018	Definity^®^ microbubbles	InSightec^®^, Haifa, Israel	Alzheimer’s Disease	NCT02986932
6 months	November 2018–December 2020	Unknown	InSightec^®^, Haifa, Israel	Alzheimer’s Disease	NCT03739905
5 years	September 2018–December 2024	Unknown	InSightec^®^, Haifa, Israel	Alzheimer’s Disease	NCT03671889
6 months	April 2020–December 2020	Definity^®^ microbubbles	InSightec^®^, Haifa, Israel	Alzheimer’s Disease	NCT04526262
1 day	December 2020–December 2021	Definity^®^ microbubbles	InSightec^®^, Haifa, Israel	Amyotrophic Lateral Sclerosis	NCT03321487
2 weeks	November 2018–December 2021	Luminity^®^ microbubbles	InSightec^®^, Haifa, Israel	Parkinson’s Disease	NCT03608553
1 day ^1^	January 2015–July 2021	Definity^®^ microbubbles	InSightec^®^, Haifa, Israel	Brain tumors	NCT02343991
~26 weeks	October 2018–December 2021	Definity^®^ microbubbles	InSightec^®^, Haifa, Israel	Glioblastoma	NCT03712293
~52 weeks	August 2018–December 2024	Definity^®^ microbubbles	InSightec^®^, Haifa, Israel	Glioblastoma	NCT03616860
~52 weeks	June 2018–December 2024	Definity^®^ microbubbles	InSightec^®^, Haifa, Israel	Glioblastoma	NCT03551249
~42 weeks	October 2018–Mar 2022	Definity^®^ microbubbles	InSightec^®^, Haifa, Israel	Breast cancer/Brain metastases	NCT03714243
45 days ^2^ (2 weeks)	August 2018–June 2019	Sonovue^®^	NaviFUS^®^, Tapei City, Taiwan	Recurrent Glioblastoma	NCT03626896
38 weeks	June 2020–December 2022	Sonovue^®^	NaviFUS^®^, Tapei City, Taiwan	Recurrent Glioblastoma	NCT04446416
52 weeks	October 2014–July 2018	Sonovue^®^	Carthera^®^, Paris, France	Recurrent Glioblastoma	NCT02253212
~39 weeks	April 2017–October 2020	Sonovue^®^	Carthera^®^, Paris, France	Alzheimer’s Disease	NCT03119961

^1^ The area that was sonicated by FUS was surgically resected 24 h after sonication. ^2^ The area that was sonicated by FUS was surgically resected 2 weeks after sonication.

**Table 6:** SCI benchmarking results: preferred routes of administration for the treatment of SCI.

Drug	Company/Partner	Indication	Clinical Phase	Modality	Target	Route of Administration
ABT-555 (ELEZANUMAB)	AbbVie	Acute cervical SCI	Phase 2	Antibody to inhibit the Neogenin and BMP pathways	RGMa (N-terminal)	Intravenous infusion
AXER-204	ReNetX Bio	Chronic cervical SCI	Phase 1/2	Fusion protein to Inhibit the NgR pathway	Nogo-A, MAG, and OMgp	Intrathecal Infusion
MT-3921	Mitsubishi Tanabe	Acute cervical SCI	Phase 2	Antibody to inhibit the BMP pathway	RGMa (C-terminal)	Intravenous infusion
NG-101/ATI-355	Novartis	Acute cervical SCI	Phase 2	Fc antibody fragment to inhibit the NgR pathway	Nogo-A	Intrathecal Injection

**Table 7:** Maturity and Potential Impact of “Brain-Biodistribution Enhancing” Technologies Surveyed.

Category	Sub-Category	Exo-Somes	Exo-AAVs	Nano-Primers	FUS (Focused Ultrasound)	CED (Convection Enhanced Delivery)	ICV (Intra-Cerebro-Ventricular)	IT (Intra-Thecal)
Potential medical impact (Null Hypothesis)	Preclinically enabling	y	y	y	y	y	y	y
	Medically enabling	y	y	TBD	y	y	y	y
	Medically transformative	TBD	n	n	n	TBD	y	n
Extent of brain tissue exposure		All brain tissues	All brain tissues	n/a	Inherently limited	Limited to targeted area	All brain tissues	Spinal tissues
Technology maturity	Readiness for Phase 1/2	y	n	n	Phase I only	y	y	y
	Safety (Chronic)	TBD	TBD	Potential secondary tox	TBD	Y as implant	Y as implant	Y as implant
	Safety (Short term)	y	TBD	Potential secondary tox	y	y	y	y
	Regulatory pathway	y	TBD	Nanoparticle predicate	n	y	y	y
	Invasiveness/medical benefit ratio (Chronic treatment)	n/a	n/a	Non-invasive	Non-invasive FUS, micro-bubbles are invasive (n)	y	y	y
	Invasiveness/medical benefit ratio^x^ (Short-term treatment)	n/a	n/a	Non-invasive	Non-invasive FUS, micro-bubbles are invasive (n)	y	y	y
	Adoption prospect by patient & care givers & payers	No foreseeable issue	No foreseeable issue	No foreseeable issue	n	y with design modification	y	y
Business impact (Null hypothesis)	Large markets/Primary indications	Stroke, potentially others	Too early to tell	Too early to tell	n	n	n	y
	Niche markets/Secondary indications	Others	Too early to tell	Too early to tell	y	y	y	y
	R&D productivity/Preclinical studies	y	y	y	y	y	y	y
Potential threat in Neuro-science	Rate of evolution	Rapidly advancing clinical stage	Very early, slow progress	Rapidly advancing POC stage	Low	Steady advancement	Significant recent advances	Low
	Competitive risk	High	Low	Low	Low	Medium	High in niche areas	High
Neuroscience assets that may benefit		Biologics, degraders	AAV-delivered therapeutics	Payload- carrying nanoparticles	All therapeutics	All therapeutics	All therapeutics	Treatment of SCI

## Data Availability

Not applicable.
